# Antimicrobial Susceptibility Patterns of *Salmonella enterica* Serotype Typhi in Eastern Nepal

**Published:** 2007-03

**Authors:** Basudha Khanal, Sanjib Kumar Sharma, Shyamal Kumar Bhattacharya, Narayan Raj Bhattarai, Monorama Deb, Reba Kanungo

**Affiliations:** Department of Microbiology, B.P. Koirala Institute of Health Sciences, Dharan, Nepal

**Keywords:** *Salmonella* Typhi, Typhoid, Drug resistance, Microbial, Microbial sensitivity tests, Ciprofloxacin, Minimum inhibitory concentrations, Nepal

## Abstract

The aim of the present study was to evaluate antimicrobial susceptibility patterns with special reference to multidrug resistance, susceptibility to ciprofloxacin, and bacteriophage typing of *Salmonella enterica* serotype Typhi isolated from blood sent for culture in a tertiary-care teaching hospital in eastern Nepal during January 2000–December 2004. In total, 132 strains of *S. enterica* Typhi, isolated from 2,568 blood culture samples collected from cases of suspected enteric fever, were tested for susceptibility to commonly-used antimicrobials by the disc-diffusion method. There were 35 multidrug-resistant strains. None of the isolates were resistant to ciprofloxacin. Of 52 isolates tested for minimum inhibitory concentration (MIC) of ciprofloxacin, 36 (69.23%) showed reduced susceptibility (MIC ≥0.25 mg/L). Of 112 strains tested for nalidixic acid susceptibility, 86 (76%) were resistant. Strains with reduced susceptibility to ciprofloxacin and resistance to nalidixic acid could be correlated. The commonest phage type was E1. Nalidixic acid susceptibility could be a useful screening test for the detection of decreased susceptibility of *S*. Typhi to ciprofloxacin, a drug which is commonly used even for minor ailments in this area.

## INTRODUCTION

Typhoid fever, a severe systemic illness, caused by *Salmonella* enterica serotype Typhi, is still an important public-health problem in many developing countries, including Nepal. According to a recently-revised global estimate, it causes 21.6 million illnesses every year, resulting in 216,500 deaths (1).

Multidrug-resistant *S*. Typhi (MDRST) is epidemiologically defined as strains resistant to any two antimicrobials in vitro even if the antimicrobials tested are known to be clinically ineffective (2). A more useful definition of MDRST is reserved for strains resistant to all three first-line antityphoidal antimicrobial agents, namely ampicillin, chloramphenicol, and trimethoprim-sulphamethoxazole (2).

Typhoid fever, caused by MDRST, has become a significant cause of morbidity and mortality over recent years. These strains have also caused outbreaks throughout the world, especially in South America, Indian subcontinent, Africa, and South-East Asia (3, 4). The incidence of MDRST is reported to be as high as 60%, although there are some reports noting its decline (5–8).

With the emergence of MDRST, fluoroquinolones have gained importance for the treatment of enteric fever in recent years. There are reports of prolonged defervescence after ciprofloxacin therapy (9–12). Strains, isolated from these cases, exhibit susceptibility to ciprofloxacin in disc-diffusion testing, but minimum inhibitory concentrations (MICs) for these strains are about 10 times higher than fully-susceptible strains. This reduced susceptibility is probably the cause for poor clinical response to treatment. Isolates of *S*. Typhi with decreasd susceptibility to ciprofloxacin have been found to be nalidixic acid-resistant. These nalidixic acid-resistant *S*. Typhi (NARST) require higher concentrations of ciprofloxacin for inhibition (4, 9, 10).

A considerable variation has been noted in the antimicrobial susceptibility patterns among isolates of *S*. Typhi as suggested in various studies conducted in different geographical locations (7, 11–17). Knowledge of the prevalence of *S*. Typhi and their antimicrobial susceptibility patterns is of utmost importance in the institution of appropriate antimicrobial therapy. Resistant *Salmonellae* and failure of ciprofloxacin therapy have been matters of concern in the reports of some studies conducted in central Nepal (8, 18–20). Phage type is an important epidemiological marker to study the movement of organisms and their spread from one geographic locale to another. Data on the susceptibility patterns and phage type prevalence of *S*. Typhi isolates in eastern Nepal are lacking. The present study was, therefore, undertaken to determine the antimicrobial susceptibility patterns of local isolates of *S*. Typhi with special reference to their multidrug resistance and reduced susceptibility to ciprofloxacin and bacteriophage types.

## MATERIALS AND METHODS

Blood samples for culture were obtained from patients who presented to the B.P. Koirala Institute of Health Sciences (BPKIHS) hospital, a tertiary-care teaching hospital in eastern Nepal, with a history of fever of variable duration from January 2000 to December 2004. Brain-heart infusion (BHI) broth, which establishes the growth of all common pathogens causing bacteraemia/septicaemia was used as a culture medium. Collection of blood, incubation, and subculture(s) onto blood agar and MacConkey agar were done as per the standard methods (21). Suspected non-lactose-fermenting colonies were further processed and identified by biochemical reactions and confirmed by group and type-specific *Salmonella* antisera (Murex Biotech, England)

Antimicrobial susceptibility was determined by the Kirby-Bauer disc-diffusion method performed on Muller-Hinton agar plates against ampicillin (10 μg), ceftriaxone (30 μg), chloramphenicol (30 μg), ciprofloxacin (5 μg), co-trimoxazole (25 μg), gentamicin (10 μg), and nalidixic acid (30 μg) (Hi Media Laboratory Ltd., Mumbai, India) (22).

The nalidixic acid-susceptibility test for *Salmonella* was introduced in our centre in 2001; thus, only *S*. Typhi isolated from 2001 onwards were tested for it.

The disk strength and zone-size interpretation was in accordance with the National Committee for Clinical Laboratory Standards (NCCLS) (22).

MIC of ciprofloxacin to 24 of 26 nalidixic acid-susceptible *S*. Typhi (NASST) and 28 randomly-selected NARST was determined by the agar-dilution method as per the NCCLS standards (23).

Fifty-four strains of *S*. Typhi, isolated till mid-2002, were sent to the National Phage Typing Centre, Lady Hardinge Medical College, New Delhi, for phage typing and biotyping. Strains isolated after that period could not be sent.

## RESULTS

In total, 132 strains of *S*. Typhi were isolated from 2,568 blood culture samples submitted to the Microbiology Unit of Clinical Laboratory Services, BPKIHS, from patients of suspected enteric fever. The isolates were from all age-groups, the median age being 19 years. The male-to-female ratio was 2.1:1.

Table 1 shows the antimicrobial susceptibility patterns of *S*. Typhi. Of the 132 isolates, 35 (26.4%) were MDR showing simultaneous resistance to ampicillin, chloramphenicol, and co-trimoxazole which are first-line antityphoidal drugs. The decreasing trend of MDRST was observed in 2004 (p=0.04, Cochran Armitage trend test). No isolates were resistant to ciprofloxacin and ceftriaxone. Nalidixic acid susceptibility testing was done for 112 isolates, of which 86 (76%) were resistant.

**Table 1.** Resistance patterns (%) of *S.* Typhi isolates (n=132)

Antimicrobial	2000 (n=20)	2001 (n=30)	2002 (n=25)	2003 (n=34)	2004 (n=23)	Total (n=132)
Ampicillin	7 (35)	13 (43.3)	8 (32)	11 (32)	1 (4.34)	40 (30)
Co-trimoxazole	7 (35)	10 (33.5)	6 (24)	12 (35.29)	1 (4.34)	36 (27)
Chloramphenicol	7 (35)	11 (36.6)	6 (24)	11 (32.35)	1 (4.34)	36 (27)
Ciprofloxacin	0	0	0	0	0	0
Ceftriaxone	0	0	0	0	0	0
Nalidixic acid	NT	23 (76.6)	19 (76)	28 (82.3)	16 (69.5)	86 (77)
Gentamicin	0	1 (3.33)	0	1 (2.94)	0	2 (1.5)
Multidrug resistance (ampicillin+co-trimoxazole +chloramphenicol)	7 (35)	10 (33.3)	6 (24)	11 (32)	1 (4.35)	35 (26)

Figures in parentheses indicate percentages;

NT=Not tested

MIC of ciprofloxacin was determined for 52 isolates, 24 NASST, and 28 NARST. Sixteen (30.67%) had MIC <0.25 mg/L, and 36 (69.23%) had ≥0.25 mg/L. Seven isolates with 0.25 mg/L, 14 with 0.5 mg/L, and seven with one mg/L were resistant to nalidixic acid. Eight isolates with decreased ciprofloxacin susceptibility were sensitive to nalidixic acid (Table 2).

**Table 2.** Results of nalidixic acid-screening test and MIC of ciprofloxacin

NA susceptibility disc-diffusion test (n=52)	MIC of ciprofloxacin						
	0.015 mg/L	0.03 mg/L	0.06 mg/L	0.12 mg/L	0.25 mg/L	0.5 mg/L	1 mg/L
NASST (24)	5	8	1	2	5	3	0
NARST (28)	0	0	0	0	7	14	7
Total	5	8	1	2	12	17	7

MIC=Minimum inhibitory concentration;

NA=Nalidixic acid;

NASST=Nalidixic acid-susceptible *Salmonella* Typhi;

NARST=Nalidixic acid-resistant *Salmonella* Typhi

Phage typing was done for 54 isolates of *S*. Typhi. The majority (n=35, 64.8%) of the isolates were of phage type E1, and the remaining isolates belonged to different phage groups: A (6), UVS1 (2), UVS2 (2), and D1 (1). Six were Vi-negative, and one had degraded Vi. Of the 35 E1 strains, 20 were MDR. Of the 35 MDR strains, 22 were phage-typed, of which 20 belonged to the E1 type. Forty-nine (90.5%) strains were biotype 1, and five strains (9.5%) were biotype II.

## DISCUSSION

Enteric fever is a major public-health problem in developing countries, including Nepal.

Prompt institution of appropriate antimicrobial therapy can reduce morbidity and mortality associated with this illness. Since 1948, chloramphenicol had been the mainstay of treatment of enteric fever until 1972 when chloramphenicol-resistant typhoid fever became a major problem. Outbreaks of typhoid fever occurred frequently in Mexico, India, Viet Nam, Thailand, Korea, and Peru. Although initially susceptible to ampicillin and co-trimoxazole, *S*. Typhi strains resistant simultaneously to all the first-line antityphoidal drugs emerged in the 1970s. Since then, these MDR strains have spread to Mexico, India, and other regions in an epidemic form and have rapidly emerged worldwide (4, 6). In the present study, the number of MDRST among the total isolates of *S*. Typhi was 35 (26.5%) which is high and is comparable with other reports from the Indian subcontinent (3–5). Some studies have, however, reported its decline (6–8). In the present study too, a similar pattern was observed. The number of MDR strains had declined towards the end of 2003 and continued through 2004, and this decreasing trend was statistically significant (p=0.04).

The phage type E1 appeared to be the dominant type in our series. It was also observed that 20 of the 22 MDR strains belonged to the phage type E1. These findings are concordant with reports from neighbouring India, suggesting cross-over of strains due to human traffic between the two countries (6, 11, 13–15).

With the emergence of MDR *S*. Typhi, quinolone, particularly fluoroquinolones, has been widely used and recommended as an alternative drug for typhoid fever where the first-line drug is no longer in use. Fluoroquinolones, available since the 1980s, have good in-vitro susceptibility and in-vivo efficacy against *Salmonellae,* including *S*. Typhi. Nalidixic acid, the prototype and the first member of the quinolone group, is now seldom used due to the emergence of resistant serotypes of *Salmonella.* It has also been observed that nalidixic acid-resistant *S*. Typhi has decreased susceptibility to fluoroquinolones. The clinical response to these agents in patients infected with NARST is greatly inferior to the response in those infected with nalidixic acid-susceptible strains. The prolonged defervescence or treatment failure in typhoid fever associated with ciprofloxacin and other fluoroquinolone therapy have been reported from many centres (9, 10–12, 24, 25). Since 1993, *S*. Typhi with decreased susceptibility to ciprofloxacin has been isolated with an increasing frequency in Viet Nam (4, 11). Subsequently, an extensive outbreak of NARST with decreased susceptibility to ciprofloxacin occurred in Tajikistan (26). Threlfall *et al*. observed that isolation of *S*. typhi with reduced susceptibility to ciprofloxacin increased from 0.9% in 1991 to 23% in 1999 in the Central Public Health Laboratory, UK. Most of those isolates were from patients returned recently from the Indian subcontinent, particularly India and Pakistan (9). This finding is similar to the reports from various series, suggesting that these strains are endemic in several countries of the Indian subcontinent (4, 6, 10, 25, 27).

The current MIC breakpoints for Enterobacteriaceae, including *S. enterica* for ciprofloxacin, are <1 mg/L and ≥4 mg/L for susceptibility and resistance respectively. *S*. Typhi with reduced susceptibility to ciprofloxacin has higher than normal MIC to this antimicrobial agent but is still considered susceptible by the NCCLS interpretative criteria (23, 24).

In the present study, 76% of the isolates were nalidixic acid-resistant, and all appeared to be susceptible to ciprofloxacin in disc-diffusion testing. MIC of ciprofloxacin for these NARST tested ranged from 0.25 to 1 mg/L. It demonstrates that *S*. Typhi, less susceptible to ciprofloxacin, are prevalent in our area. Similar findings have been reported from studies conducted in central Nepal (8, 18, 19).

The requirement for higher concentrations of ciprofloxacin for the inhibition of *S*. Typhi could be due to the overuse of ciprofloxacin in the treatment of typhoid and in other unrelated infections. Over-the-counter availability of antibiotics and incomplete treatment due to many reasons in a developing country like ours may also be the factors contributing to the development of resistance.

Single-point mutation in quinolone resistance-determining region of the topoisomerase gene gyrA in *Salmonella* usually leads to simultaneous resistance against nalidixic acid and decreased susceptibility to ciprofloxacin (4, 24, 28–30). Nalidixic acid resistance has been validated as a screening test for reduced susceptibility to ciprofloxacin. Identification of resistance to nalidixic acid by disc-diffusion provides a sensitive indicator of low-level resistance to ciprofloxacin. The NCCLS currently recommends the testing of extra-intestinal *Salmonella* isolates for reduced susceptibility to fluoroquinolones (31). However, there are strains of *S*. Typhi with decreased susceptibility to ciprofloxacin but susceptible to nalidixic acid. Detection of a number of such strains in the present study deserves a mention. A similar finding was observed in an Indian study and also in an European study (6, 32).

All the isolates, including NARST strains, were susceptible to ceftriaxone, although ceftriaxone-resistant *S*. Typhi was reported elsewhere (33). This finding may be important for considering the use of this antibiotic for treating infection with MDR *S*. Typhi resistant to ciprofloxacin in this region. It is also of paramount importance to limit its therapeutic use to only when the first-line and second-line drugs fail to evoke a satisfactory response or when the isolate is resistant to nalidixic acid. High cost, need for parenteral administration, and poor penetration of cephalosporin into the infected cells, thus, leading to the requirement of a prolonged course are the major limiting factors, despite being the drug of choice for the treatment of quinolone-resistant typhoid fever.

Over the recent years, azithromycin has also been used as the option for the treatment of MDR and quinolone-resistant typhoid fever (4, 34). However, its high cost and limited availability in endemic areas are again the major disadvantages.

Activity of antimicrobials on *S*. Typhi appears to be changing over the past decade. From nalidixic acid to ciprofloxacin, development of resistance has steadily progressed. Although not a major problem at present, there are sporadic reports of ceftriaxone-resistant *S*. Typhi (33). Moreover, detection of integrons in isolates of *S*. Typhi from Asia has suggested that this organism has the potential for the acquisition of new resistant genes (35, 36). Concerns have been raised about the emergence of effectively untreatable typhoid in the developing nations if the appropriate control measures are not taken on time (4, 34). The prudent use of antibiotics and effective hospital infection-control practice may play a crucial role in preventing the emergence and spread of resistant organisms. At this point of time, it is also important to consider the necessity of development and evaluation of newer antimicrobials with different target site and mechanisms of action as the need for an alternative drug becomes evident.

In conclusion, the findings of the present study indicate that MDR strains prevail in eastern Nepal. *S*. Typhi with reduced susceptibility to ciprofloxacin has emerged, thus narrowing the spectrum of availability of antibiotics for the treatment of this infection, often endemic sometimes taking epidemic proportions. Resistance of nalidixic acid as a screening test for detecting reduced susceptibility to the quinolone group of drugs merits consideration. This simple disc-diffusion test can predict probable treatment failures and, thus, obviate the need for time-consuming dilution tests. Determination of MIC is still useful not only to detect the exact inhibitory concentration but also to confirm the less-susceptible strains which may indicate the development of impending resistance among the local isolates. It is also important that microbiology laboratories have to decide on the optimal approach for timely detection of resistant strains depending upon their workflow and available resources.

This laboratory-based study highlights the patterns of *S*. Typhi in a hospital set up in eastern Nepal. As the majority of typhoid fever patients are treated on a outpatient basis, a detailed information on the presenting features, time for defervescence, and response to therapy could not be obtained. Therefore, on the basis of observations made in this study, a further surveillance on typhoid fever and its clinical and epidemiological correlation with the resistant and susceptible strain is recommended.

## ACKNOWLEDGEMENTS

The study was supported by the B.P. Koirala Institute of Health Sciences, Dharan. The authors thank Dr. Gita Mehta, Director Professor and Head, Department of Microbiology, Lady Hardinge Medical College, New Delhi, India, for phage typing the strains in National Salmonella Phage typing Centre.
